# Using High‐Resolution Radiotracking to Improve Inference About the Spatial Ecology of Small, Slow‐Moving Ectotherms

**DOI:** 10.1002/ece3.72137

**Published:** 2025-10-06

**Authors:** Kristen E. Liles, Kenia Barajas‐Salazar, Ivana Mali

**Affiliations:** ^1^ Department of Biological Sciences North Carolina State University Raleigh North Carolina USA; ^2^ Department of Forestry and Environmental Resources North Carolina State University Raleigh North Carolina USA

**Keywords:** box turtles, home range, movement, survey bias

## Abstract

Ornate box turtle ( *Terrapene ornata* ) and eastern box turtle ( *Terrapene carolina carolina* ) are long‐lived, relatively small, and slow‐moving ectotherms experiencing range‐wide declines primarily due to habitat loss. Understanding home range and movement patterns of box turtles is crucial for conservation efforts in fragmented landscapes. Very High Frequency (VHF) radio transmitters are commonly used to locate box turtles, but sample size and availability of personnel can limit how often each turtle is tracked. In this study, we evaluated whether the rate of tracking events affects the estimated home range and average daily movement of *T. ornata* in a short‐grass prairie located in Roosevelt County, New Mexico, and *T. c. carolina* in an urban forest located in Wake County, North Carolina, USA. We tracked three *T. ornata* and four *T. c. carolina* four to 7 days per week during their active season. Using the full dataset, we first calculated 100% Minimum Convex Polygon for each turtle. Then, we randomly sampled the full dataset, representing scenarios where turtles were tracked from one to four times per week, and recalculated home ranges. We also calculated distances between consecutive locations in the full dataset to evaluate how rates of tracking events affect estimates of mean daily movement. Mixed effect models revealed that home range size estimates significantly decreased with less frequent tracking events. Furthermore, we found that turtles occasionally moved longer distances within a single day. Our findings suggest that these rare bursts of movement are ecologically relevant but may be overlooked, and consequently home range sizes underestimated, if turtles are not tracked frequently.

## Graphical abstract

This research shows that radio‐tracking schedules affect home range estimation in North American box turtles. Occasionally, turtles moved long‐distances in a single day. These rare movements are important but can be missed without frequent tracking.

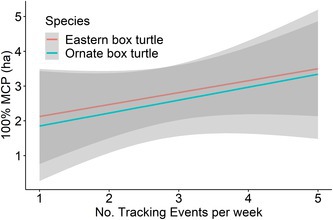

## 1 Introduction

**FIGURE 1:**
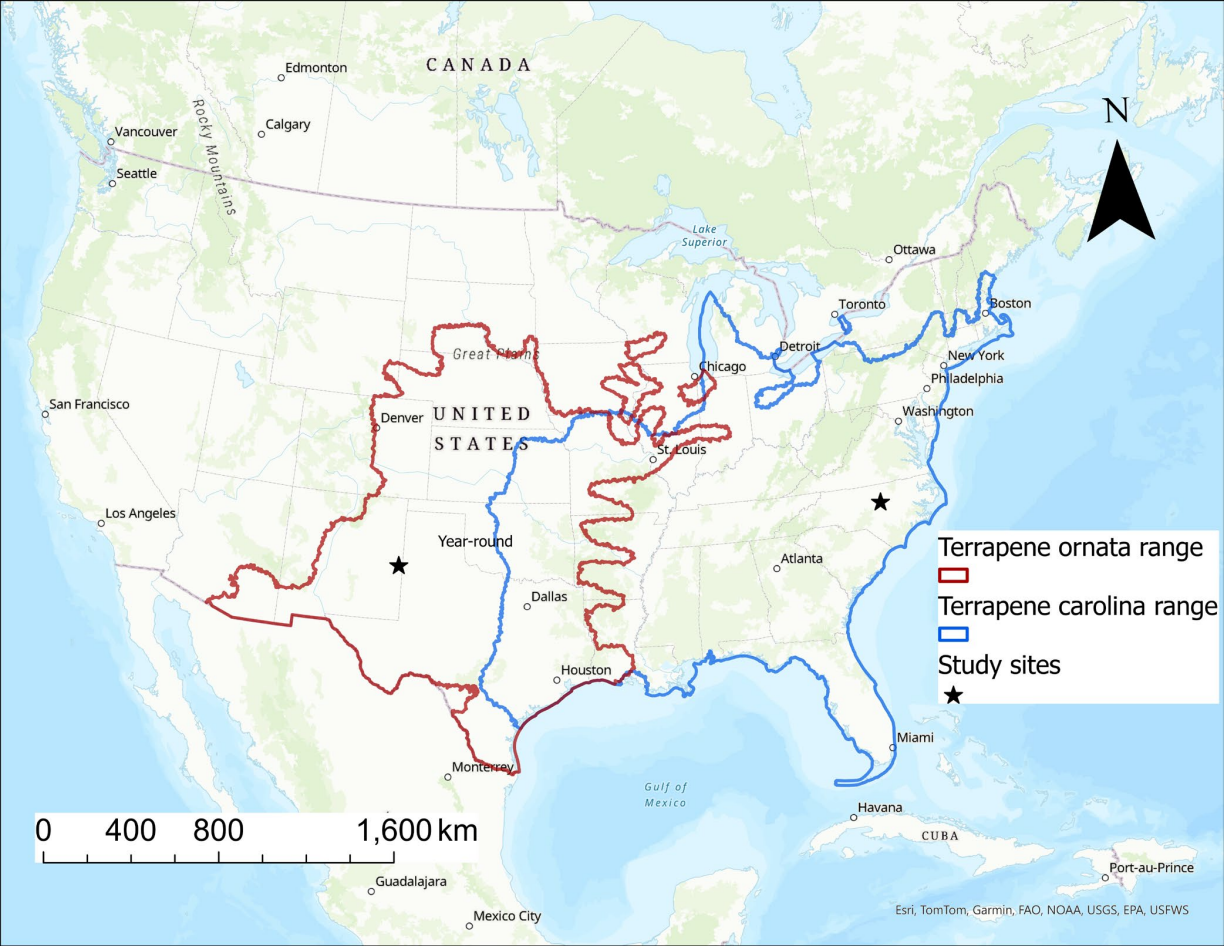
A map of North America, outlining the distribution of *Terrapene ornata* and *Terrapene carolina* in the USA (downloaded from the United States Geological Survey) and the study sites located in Roosevelt County, New Mexico and Wake County, North Carolina. Note that recent taxonomic changes recognized several species within the *T. carolina* complex.

Studying animal movement patterns and use of available habitat across time and space is essential for understanding the behavior and ecology of a species (Vannatta and Klukowski 2020; Hjort et al. 2021). Moreover, this information is often incorporated into local wildlife management decisions, broad conservation plans, species status assessments, and recovery plans (Fraser et al. 2018; Martin and Root 2020; Roe et al. 2021; Hulbert et al. 2024). Monitoring movement is usually accomplished by tracking individual animals within a population. Larger amounts of data (i.e., more location points) with high location accuracy is more desirable as they can provide better information on the animals' behavior and microhabitat use. North American box turtles (Genus *Terrapene*) are relatively small, long‐lived terrestrial ectotherms with slow moving speeds (Ernst and Lovich 2009). Traditionally, two species were thought to occur in the United States of America (USA) and have wide distributions: *Terrapene ornata* and *Terrapene carolina* (Ernst and Lovich 2009; Figure 1). Today, there are several recognized subspecies and new species level recognitions still in flux (Turtle Taxonomy Working Group 2021). The behavior and activity of box turtles are strongly influenced by geography and local environmental conditions due to their ectothermic nature (Ernst and Lovich 2009). Box turtle movement and home range have been studied extensively. Yet, the high‐resolution data that would enable better understanding of their decision‐making to move and utilize different habitat patches is still lacking.

*Terrapene ornata* is a prairie and desert dwelling species distributed across central USA, from southern Wyoming to Indiana and across southern Louisiana and southern Arizona (Ernst and Lovich 2009; Figure 1). It inhabits dry, short vegetative habitats with extreme temperature fluctuations, and consequently, it has a bimodal activity pattern to escape the hot and dry mid‐day temperatures (Converse 1999; Converse and Savidge 2003; Tucker et al. 2015; Suriyamongkol et al. 2021). Its eastern counterpart, *T. carolina* , is primarily a forest dwelling species associated with a more humid climate and distributed from southern Maine to northern Florida and across to parts of southern Illinois, northern Mississippi, Kentucky, and Tennessee (Ernst and Lovich 2009; Figure 1). For both species, the increase in activity has been attributed to precipitation events (Reagan 1974; Stuart and Miller 1987; Dodd Jr. et al. 1994). While *T. ornata* does not need standing water to survive, this species has been found submerged in edges of ponds (Kosmicki et al. 2024). *Terrapene carolina* is often found near creeks and wetlands (Roe et al. 2018). Some *T. c. carolina* are known to migrate to wetlands in the hot summer months (Donaldson and Echternacht 2005), while *T. carolina major* is more permanently associated with aquatic habitats like forested wetlands (Meck et al. 2020).

A wide range of methods have been used to study *T. ornata* and *T. carolina* movement patterns and home range, including Very High Frequency (VHF) radio telemetry, thread trailing, visual encounter capture‐mark‐recapture surveys, and more recently GPS tags, each having its strengths and weaknesses (Stickel 1950; Donaldson and Echternacht 2005; Vannatta and Klukowski 2020; Hulbert et al. 2024). The most commonly used approach has been and continues to be VHF radio telemetry (Ribeiro et al. 2024). Thread‐trailers are often used in conjunction with VHF transmitters to give detailed insight into short‐term travel routes and assist in finding nesting sites, but the thread‐trailer must be followed and replaced daily, which is labor intensive (Iglay et al. 2006; Vannatta and Klukowski 2020). In addition, the thread can snap easily, unwind completely, or get wrapped around a turtle (Breder 1927; Dodd 2001; Vannatta and Klukowski 2020). Capture‐mark‐recapture through visual encounter surveys (VES) is technologically less expensive because turtle shells are easy to mark for future identification. However, VES are labor intensive, and they are known to yield low detection rates, making it difficult to use for home range estimation, though tracking dogs have been utilized to increase the likelihood of encountering a turtle (Dodd 2001; Kapfer et al. 2012; Vannatta and Klukowski 2020). More modern approaches to understanding box turtle behavior and movement patterns involve the use of GPS tags. These devices can gather position data frequently and remotely, allowing for fine scale analyses without requiring manual labor or disturbing the turtle (Cagnacci et al. 2010; Hulbert et al. 2024). However, the current technology suggests that GPS data points may lack the accuracy needed for studying fine scale movement, and their functionality is significantly affected by weather conditions and landscape characteristics (Hulbert et al. 2024).

VHF radio‐telemetry system requires on‐foot tracking, utilizing an antenna and radio receiver to locate the turtle with an attached radio transmitter. Although highly accurate, this method is known to be labor intensive due to the time and effort it takes researchers to find and record the locations of the turtles (Harris et al. 1990; Vannatta and Klukowski 2020). To combat this, researchers often set a determined sampling protocol for tracking the turtles, which is dependent on the sample size (i.e., number of turtles tracked), availability of personnel, and funding to cover the cost of the equipment and field technicians. How often turtles are located during their active season varies among studies. Reports range from 1 to 3 times per week (Converse and Savidge 2003; Donaldson and Echternacht 2005; Rossell et al. 2006; Hester et al. 2008; Hill et al. 2009; Currylow et al. 2011; Kapfer et al. 2013; Henriquez et al. 2017; Roe et al. 2019, 2021; Harris et al. 2020; Altobelli et al. 2021; Brown et al. 2021), once every 7 to 10 days (Colbert et al. 2023), or once a month (Iglay et al. 2007; Brisbin et al. 2008). Few studies have tracked turtles on a daily or near‐daily basis (e.g., Bernstein et al. 2007; Refsnider et al. 2012; Suriyamongkol et al. 2021; Hulbert et al. 2024).

*Terrapene carolina* and *T. ornata* have significant individual variability in home ranges and mean daily movements. Based on Minimum Convex Polygon (MCP) estimations, *T. c. carolina* home ranges generally average 2 to 9 ha (Hester et al. 2008; Kapfer et al. 2013; Kiester and Willey 2015) but reported individual home ranges can be as small as 0.21 ha (Kapfer et al. 2013) and as large as 113 ha (Roe et al. 2020). The mean daily movement, calculated as the distance between two consecutive points divided by the number of days between tracking events, ranges from 8.6 m/day (Hester et al. 2008) to 24.0 m/day (Roe et al. 2020). *Terrapene ornata* home ranges average 1 to 5 ha (Holm 2003; Refsnider et al. 2012; Habeck et al. 2019), with individual home ranges being as small as 0.03 ha (Nieuwolt 1996) and as large as 30.9 ha (Weaver et al. 2025), while daily movement averages from 13.4 m/day (Nieuwolt 1996) to 35 m/day (Weaver et al. 2025). These extremely small and extremely large home range sizes are often attributed to very few individuals within a population (Donaldson and Echternacht 2005; Henriquez et al. 2017; Weaver et al. 2025). Individual box turtles have been shown to exhibit unique personality traits and behavioral patterns (Pich et al. 2019; Carlson and Tetzlaff 2020; Reed et al. 2023; Hughes et al. 2025). However, these traits are potentially related to a combination of environmental conditions and individual health (Kashon and Carlson 2018).

While sampling protocols can be helpful in creating set tracking schedules for researchers, they could also inadvertently affect home range size estimations. Comparing home ranges and mean daily movement patterns of box turtles across different studies using VHF radio tracking may not always be appropriate. The number of location points collected over study duration (e.g., the number of location points collected every week or month) could potentially affect estimations of these important spatial metrics. Researchers that locate turtles infrequently could overlook ecologically important movement events, possibly resulting in movement and home range estimations that do not depict the true extent of the utilized landscape. This possibility has often been acknowledged but never explicitly tested. In this study, we intentionally prioritized more frequent tracking of fewer individuals of *T. ornata* and *T. carolina carolina* with an objective to examine how the number of location data points influences home range and mean daily movement estimates.

## 2 Materials and Methods

### 2.1 Study Sites

We studied *T. ornata* at the Eastern New Mexico University (ENMU) Preserve in Roosevelt County, New Mexico, USA (Figure 1). The preserve is a ~121 ha wildlife study area utilized by the biology department at ENMU. The Preserve is an undisturbed grassland field, not open to the public, nor is grazing permitted. The Preserve is bordered by a state highway, private lands, and undisturbed rangeland. We studied *T. carolina carolina* at a ~102 ha multi‐use working forest in Wake County, North Carolina, USA (Figure 1). The forest is managed by North Carolina State University, open to the public, and used for research, teaching, and outdoor recreation. The forest consists of several habitat types, including coniferous forests, mixed forests, wetlands, and shrublands, and is surrounded by neighborhoods and major highways.

### 2.2 Data Collection

Between September 5th, 2019, and July 7th, 2020, we found three *T. ornata* (one female—labeled A; two males—labeled B and C) during VES. We found four *T. c. carolina* between April 24th and May 1st, 2023, two males—labeled A and C and two females—labeled B and D, while also conducting VES. We marked the turtles by notching marginal scutes (Cagle 1939) and obtained body measurements and pictures of the carapace and plastron. At the time of initial capture, the weights of turtles ranged from 277 to 341 g and 340 to 458 g for *T. ornata* and *T. c. carolina*, respectively. We then equipped turtles with VHF radio transmitters (RI‐2B, Holohil; Carp, Ontario, Canada) on their costal scute with steel‐reinforced epoxy putty. Placement of the radio transmitters followed the standard protocol, and the weight of the transmitter and the epoxy did not exceed 7% of turtle body weight.

We located the seven turtles 5 to 7 days a week. On a few occasions, primarily due to inclement weather or scheduling conflicts, we tracked *T. ornata* three to four times per week and *T. c. carolina* four times per week (with an exception of one turtle that was tracked three times 1 week due to difficulties of crossing the flooded creek). It is important to note that the overall effort (i.e., personnel hours) was equivalent to tracking ~30 individuals once a week, the most commonly reported tracking schedule in the literature. To track turtles, we used an R410 Advanced Telemetry Systems receiver (Isanti, Minnesota, USA) and a RA‐23K VHF Telonics antenna (Mesa, Arizona, USA). Data collected consisted of turtle ID (i.e., specific frequency of the transmitter), date, time, location coordinates (using handheld GPS with location accuracy of 5 m or better), turtle activity (above ground, underground/hiding, or unsure), pictures of the turtle and the surrounding habitat, and a brief written description of the turtle's behavior and surrounding habitat.

### 2.3 Home Range and Movement Estimation

We used the data collected from May 3rd to October 20th, 2021, for *T. ornata* , and May 1st to October 31st, 2023, for *T. c. carolina*, which represents the box turtle primary activity period in our two study systems. We assigned each tracking event a week, with May 3rd, 2021 (Monday) and May 1st, 2023 (Monday) representing the start of Week 1 and so on. One *T. ornata* did not emerge from brumation until Week 3 and one *T. ornata* entered brumation a week earlier, so we omitted those weeks for these specific turtles. Within each week, we assigned each day a number from 1 to 7, with Monday representing Day 1 and Sunday Day 7. In total, we collected 394 daily observations across all three *T. ornata* , ranging from 127 observations to 135 observations per turtle. Across all weeks, we mostly located turtles seven times a week (48.61% of all weeks), with three, four, five, and six tracking days per week constituting 22.22%, 15.28%, 4.17%, and 9.72% of the dataset, respectively. In total, we collected 587 daily observations across all four *T. c. carolina*, ranging from 141 observations to 148 observations per turtle. Across all weeks, we mostly located turtles five times a week (43.69% of all weeks), with four, six, and seven tracking days per week constituting 3.88%, 33.98%, and 18.45% of the dataset, respectively. One week, a single *T. c. carolina* was tracked only three times due to difficulty in accessing the turtle's area (turtle D, week 8).

To estimate home range size, we used 100% MCP assuming each location point is ecologically and biologically relevant. Although Kernel Density (KD) is often reported alongside MCP, KD may not be an appropriate estimator of home range for herpetofauna due to the significant influence of chosen smoothing parameters in these models (Row and Blouin‐Demers 2006). In addition, our study objectives did not focus on patch use or activity centers, which are primary applications of KD. For each turtle, 100% MCPs were calculated using package *adehabitat* in RStudio (Calenge 2006). First, we estimated home ranges for each turtle using all location points (127–148 data points per turtle). We then simulated the full dataset by randomly subsampling a specific number of days (*n*) from each week and reiterated the process 999 times. We repeated this process for *n* = 1, 2, 3, and 4 for *T. c. carolina* and *n* = 1, 2, and 3 for *T. ornata* , representing scenarios where turtles were tracked from one to four times per week. We calculated home ranges for each iteration and then calculated the mean home range and 95% confidence intervals (CI) for every scenario. To test the relationship between home range estimates and the number of tracking occasions per week, we used a linear mixed effect modeling framework in package *lmer* in RStudio. Means of each simulation including the home range of the full dataset represented the response variable. The number of tracking events per week and species were used as fixed effects, while individual turtles were used as a random effect to account for repeated measurements. For simplicity, we treated the home range of the full dataset as five tracking events per week.

Using all data points per turtle, we measured distances between consecutive location points using package *adehabitatLT* in R (Calenge 2006). To standardize the data in accordance with the literature, we divided the distance by the number of days between tracking events. We then divided the dataset based on the days between tracking events and created box plots to identify outliers and evaluate the differences in estimated mean daily distances moved between point locations recorded ~1 day apart and point locations recorded > 1 day apart.

## 3 Results

**FIGURE 2:**
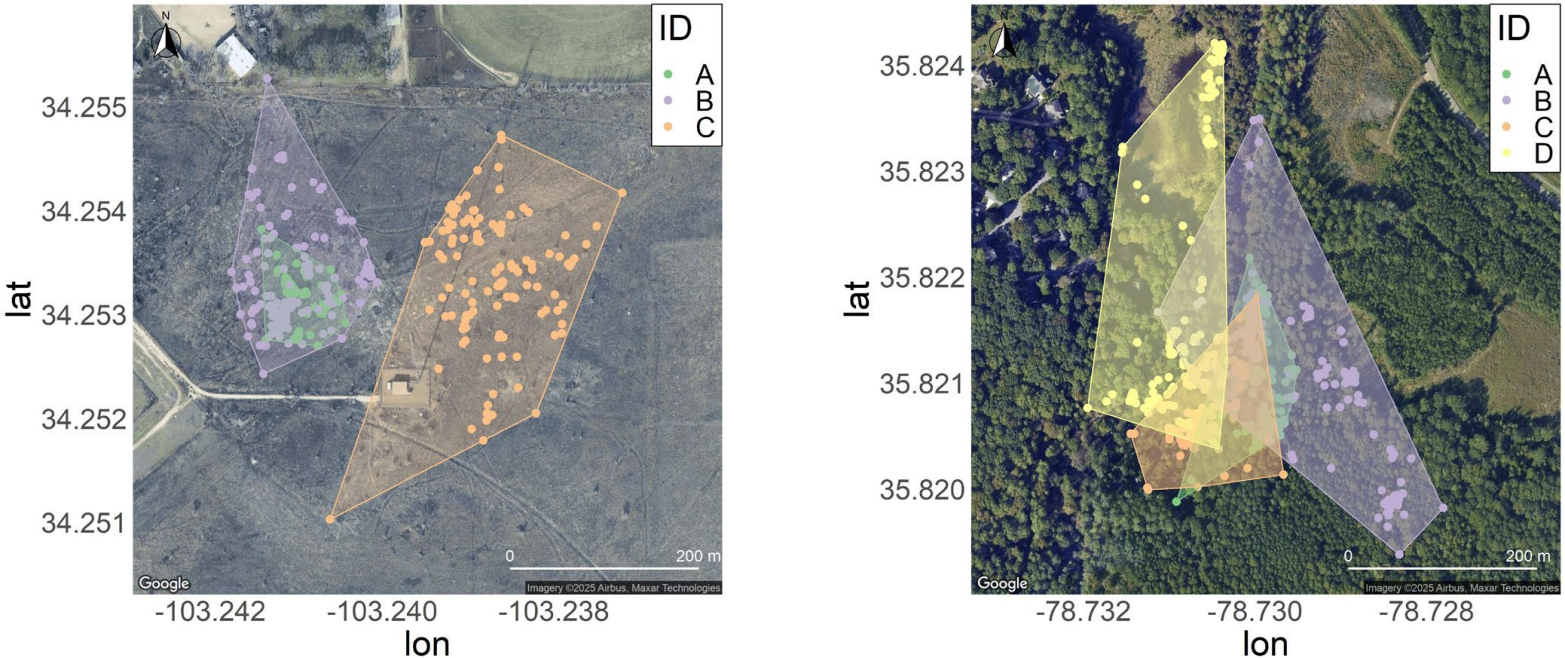
A map of individual location points and 100% MCP home range of *Terrapene ornata* (left) radio‐tracked in 2021 in Roosevelt County, New Mexico, USA, and *Terrapene carolina carolina* (right) radio‐tracked in 2023 in Wake County, North Carolina, USA, using the full datasets. Letters represent individual turtles.

**FIGURE 3:**
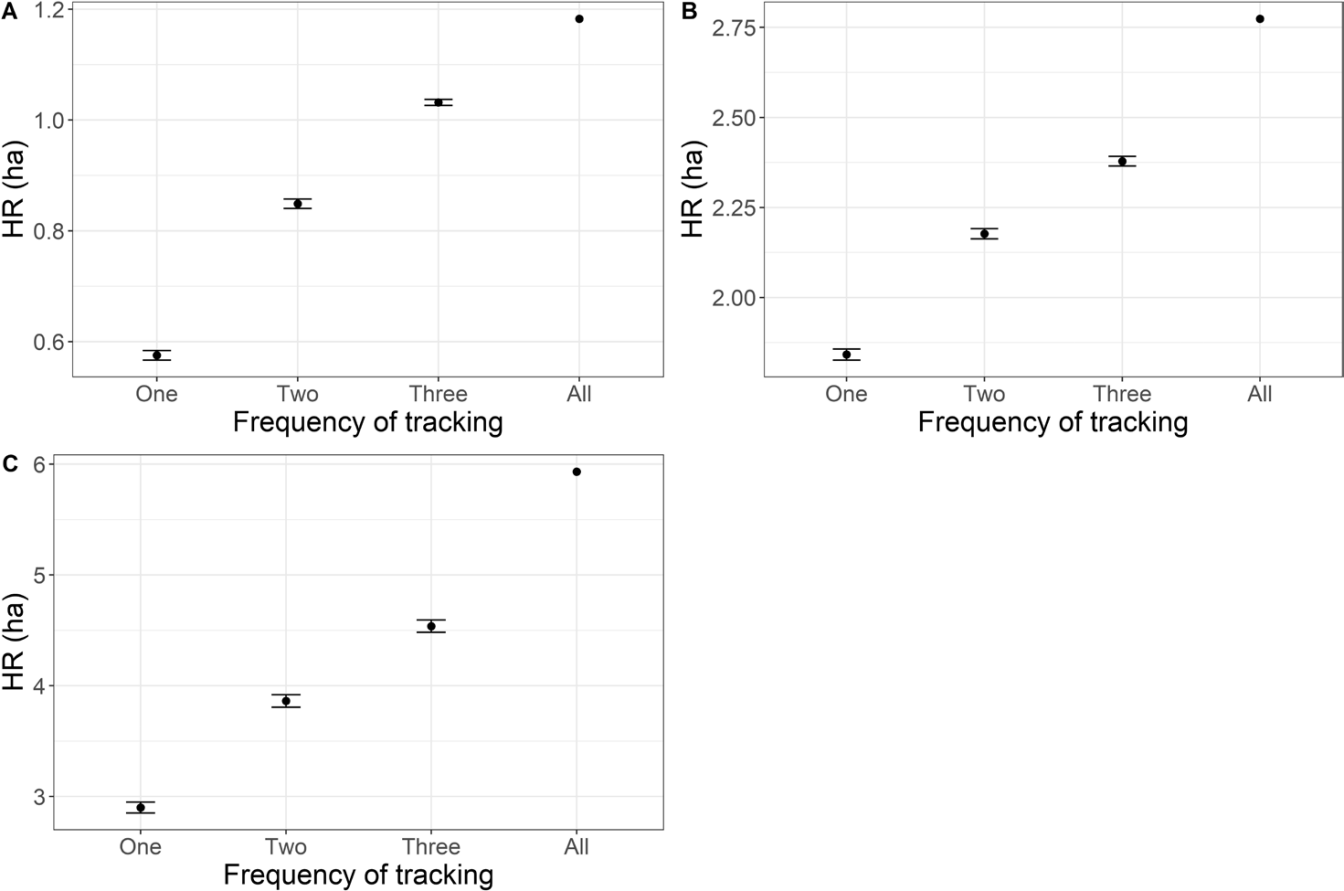
100% MCP home range size estimates (black circles) and 95% confidence intervals (bars) for each data simulation that represents different frequency of tracking (i.e., number of tracking events from One to Three times per week), and the estimate using the full data set (i.e., All) for three *Terrapene ornata* studied in Roosevelt County, New Mexico, USA, in 2021. Letters represent individual turtles.

**FIGURE 4:**
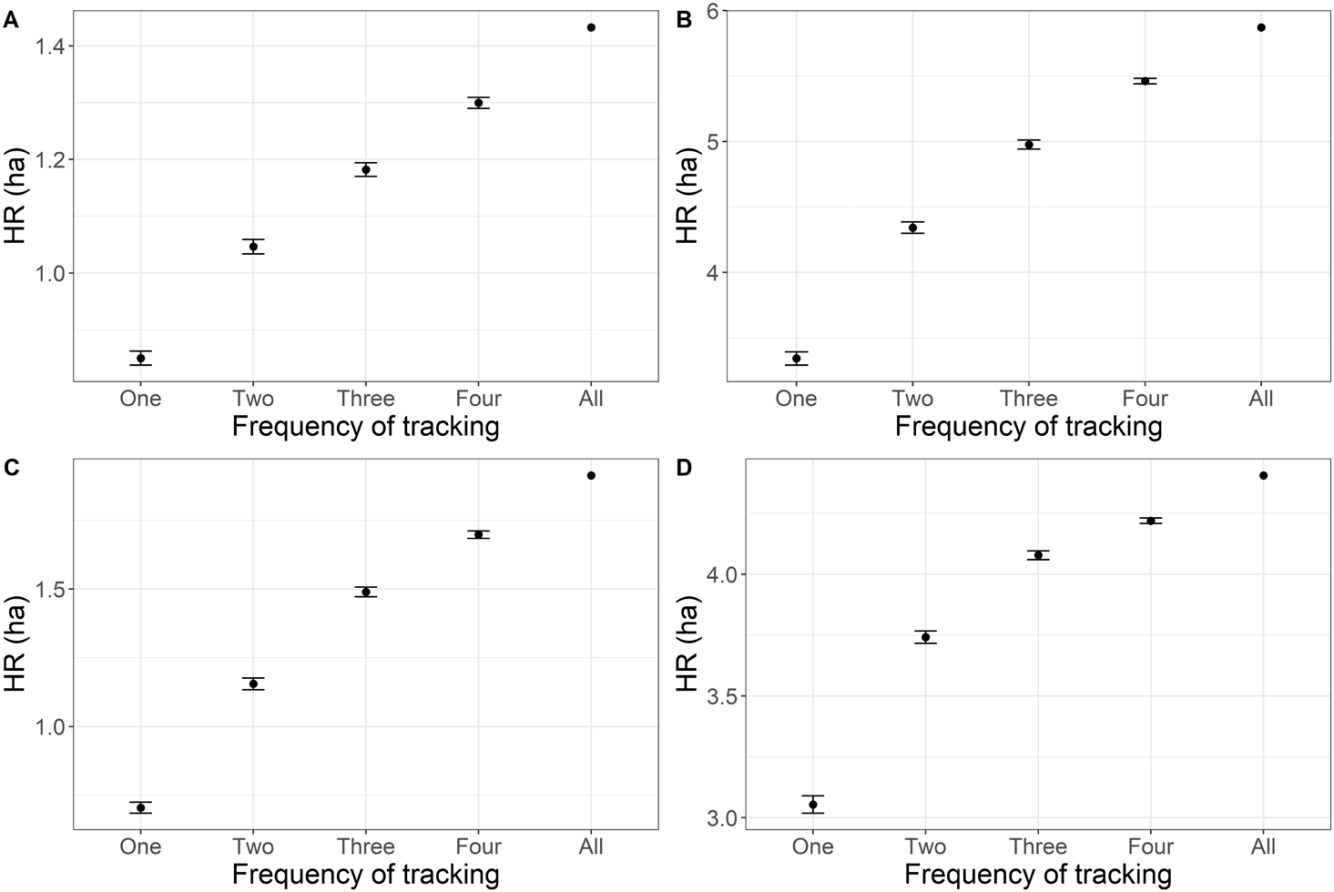
100% MCP home range (HR) size estimates (black circles) and 95% confidence intervals (bars) for each data simulation that represents different frequency of tracking (i.e., number of tracking events from One to Four times per week), and the estimate using the full data set (i.e., All) for four eastern box turtles ( *Terrapene carolina carolina* ) studied in Wake County, North Carolina, USA, in 2023. Letters represent individual turtles.

**FIGURE 5:**
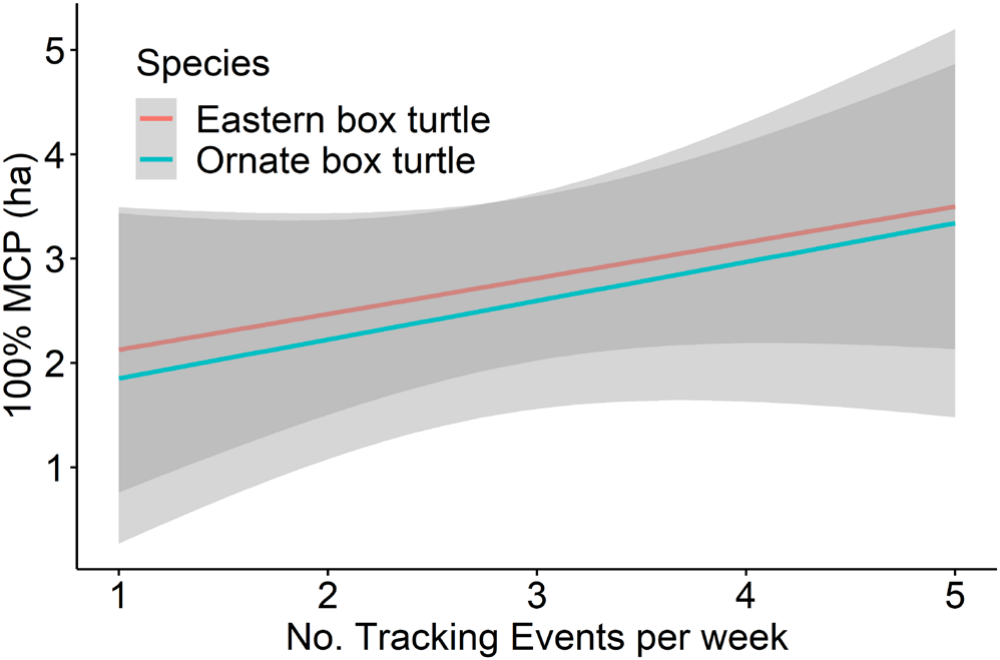
Relationship between the number of weekly tracking events and home range size (100% MCP) estimate for ornate box turtle ( *Terrapene ornata* ) and eastern box turtle ( *Terrapene carolina carolina* ) based on the mixed effect model, treating individual turtle as a random effect.

When utilizing the full dataset, the home range for *T. ornata* ranged from 1.2 to 5.9 ha, with a mean and median of 3.3 ha and 2.8 ha, respectively (Figure 2). The home range of *T. c. carolina* ranged from 1.4 to 5.9 ha, with a mean and median of 3.4 ha and 3.2 ha, respectively, when utilizing the full dataset (Figure 2). There was no significant difference in home ranges between the two species (estimate = −0.22, SE = 1.35, *p* = 0.88). There was a significant positive relationship between the number of tracking events per week and estimates of home range size (estimate = 0.36, SE = 0.05, *p* < 0.01; Figures 3, 4, 5). There was substantial variation among individuals, indicating individual‐level heterogeneity in home range size (i.e., SD of the random effect = 1.76). Based on the model, the estimated home range increased by approximately 36% for each additional day of tracking. In other words, the home range estimate was ~1.7 times larger using the full dataset than the home range estimation using one location point per week.

**FIGURE 6:**
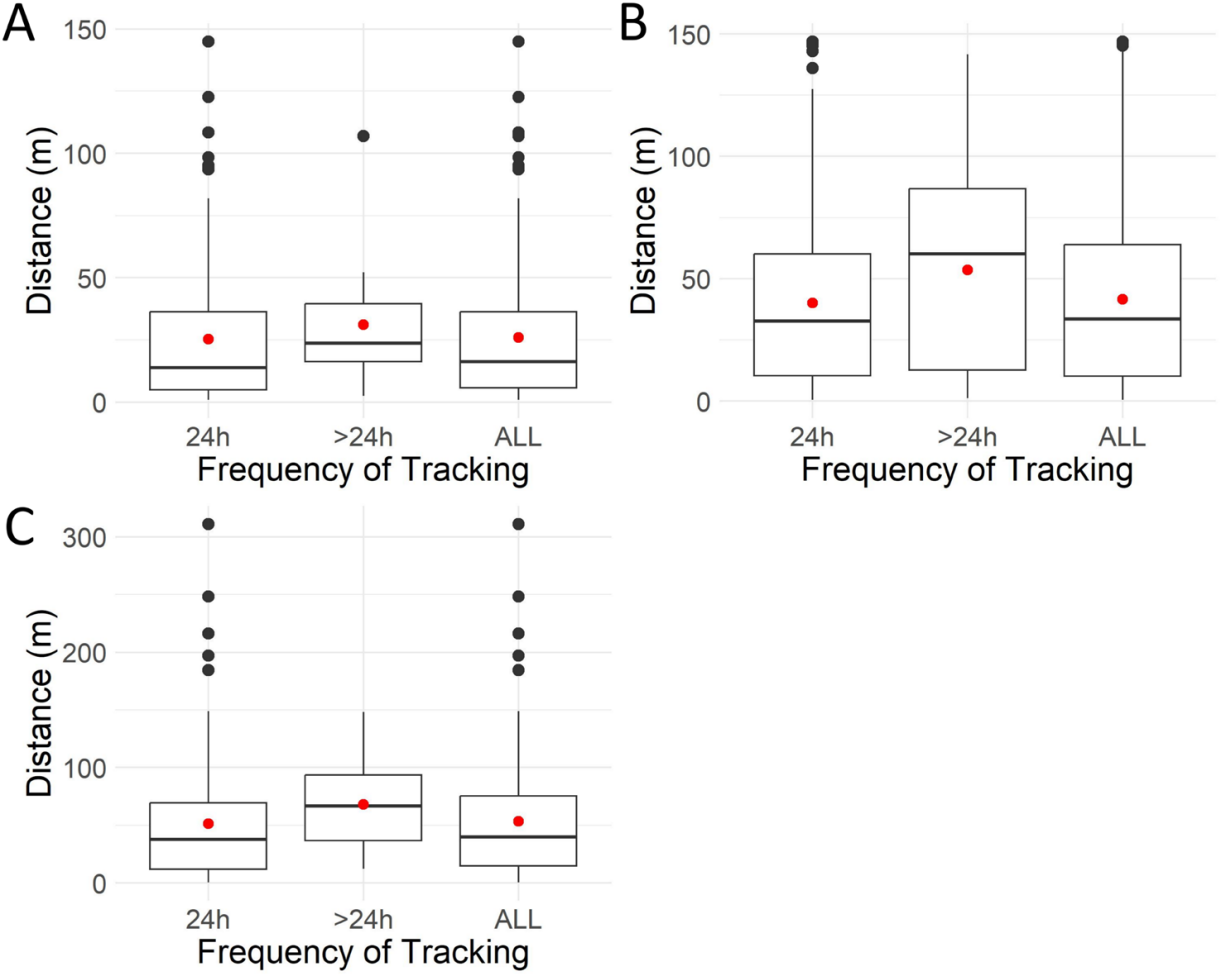
Boxplots representing daily distances moved by ornate box turtles ( *Terrapene ornata* ), broken down by frequency of tracking (i.e., 24 and > 24 h between tracking events) and with the data pulled together (ALL). Red dots represent the mean. Letters represent individual turtles.

**FIGURE 7:**
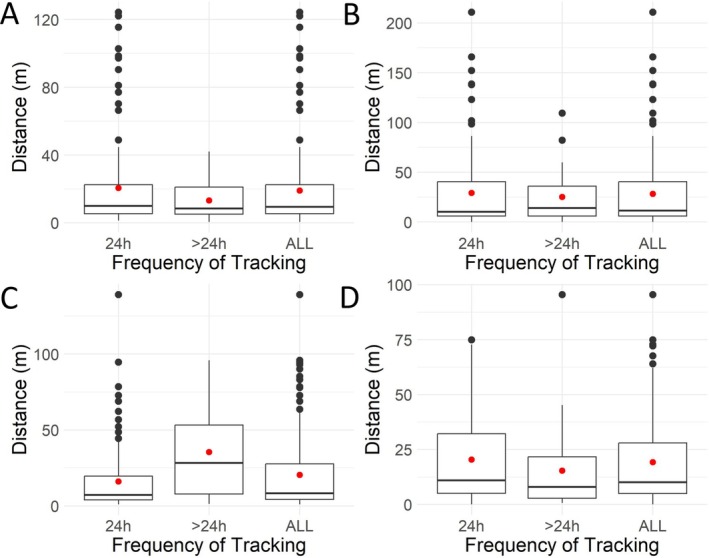
Boxplots representing daily distances moved by eastern box turtles ( *Terrapene carolina carolina* ), broken down by frequency of tracking (i.e., 24 and > 24 h between tracking events) and with the data pulled together (ALL). Red dots represent the mean. Letters represent individual turtles.

Based on the full dataset, *T. ornata* moved between 0 and 311 m per day, with the overall mean ranging from 24 to 48 m per turtle. *Terrapene c. carolina* moved between 0 and 210 m per day, with the overall mean ranging from 16 to 28 m per turtle. Mean daily movement was similar between more and less frequently obtained location points for all seven turtles; however, there were multiple outliers (i.e., movement distances outside of the interquartile range) identified within the dataset with only ~1 day between tracking events (Figures 6 and 7). Specifically, *T. c. carolina* had 31 total movement events considered outliers (1–12 per turtle), while *T. ornata* had 15 total outlier movements (4–6 per turtle; Table 1).

**TABLE 1:** The number and percentage of outlier daily movement events when ornate box turtles ( *Terrapene ornata* ) and eastern box turtles ( *Terrapene carolina carolina* ) were tracked at ~24 h intervals, divided by the month of the year. *Terrapene ornata* were radio‐tracked in 2021 and *T. c. carolina* in 2023.

Species	ID (Sex)	May	June	July	August	September	October	Total
*T. ornata*	A (F)	0	0	2 (33%)	4 (67%)	0	0	6
*T. ornata*	B (M)	0	0	2 (50%)	0	1 (25%)	1 (25%)	4
*T. ornata*	C (M)	0	1 (20%)	1 (20%)	1 (20%)	0	2 (40%)	5
*T. c. carolina*	A (M)	0	0	4 (33%)	7 (58%)	1 (8%)	0	12
*T. c. carolina*	B (F)	0	1 (12%)	3 (38%)	4 (50%)	0	0	8
*T. c. carolina*	C (M)	2 (20%)	3 (30%)	3 (30%)	0	1 (10%)	1	10
*T. c. carolina*	D (F)	0	0	1 (100%)	0	0	0	1

Based on the weather data from the nearby NOAA weather station (US1NCWK0345), of the 31 outlier movements observed in *T. c. carolina*, 20 (65%) occurred on or immediately after a rainy day. In addition, 9 (29%) occurred when rainfall was recorded at least once within the 5 days prior (including the day of), and only 2 (6%) occurred with no rain recorded in the preceding five‐day period. Turtle movements were generally random and uncorrelated, with only two individuals (A and B) moving on the same date on three separate occasions. Males exhibited more outlier movements than females (22 and 9, respectively; Table 1) and most of the outlier movements occurred in July and August (11 in each month; Table 1). The largest turtle, female D, had only one outlier movement but one of the largest overall home ranges. Interestingly, the smallest turtle in the dataset was also a female, and both females had substantially larger home ranges than males.

Based on the weather data from the nearby NOAA weather station (US1NMRV0028), of the 15 outlier movements observed in *T. ornata* , 6 (40%) occurred on or immediately after a rainy day. In addition, 9 (33%) occurred when rainfall was recorded at least once within the 5 days prior (including the day of) and 3 (27%) occurred with no rain recorded in the preceding five‐day period. All turtles exhibited a similar number of outlier movements, and no individuals moved on the same date. Most outlier movements occurred in July and August (five in each month; Table 1). Both males had larger home ranges than the female, who was the largest individual in the dataset. The extensive home range of male C was largely driven by a single outlier movement (Figure 2).

## 4 Discussion

In this study, we investigated the effects of the number of location points obtained through VHF radio tracking on home range and mean daily movement estimations of two widely distributed species of North American box turtles. Despite the abundance of studies on box turtle movement and home range, this has not been explicitly addressed before. Our overall findings suggest that the number of location points per week affects the home range size estimates with a significant positive correlation. Studies that track turtles on a weekly basis may draw estimates that are approximately half of the individual home range estimated when tracking five or more days a week. When studying movement patterns, researchers traditionally divide the distance between two consecutive locations by the number of days between the tracking events to standardize the data (Nieuwolt 1996; Weaver et al. 2025). This approach accounts for the inability to determine how movement was distributed across days when tracking occurs infrequently, which can obscure understanding of box turtle daily behavior. In our study, frequent tracking resulted in more outliers among daily movement calculations compared to less frequent tracking, although the means were similar. We found the peak of outlier events to be in the months of July and August, which may correlate with box turtle mating and nesting activities (Ernst and Lovich 2009). In combination with home range estimations, these findings demonstrate that box turtles occasionally make greater than expected movements that are unaccounted for if tracked infrequently.

We collected 127–148 location points per turtle and found the maximum annual home range size for both species to be ~6 ha, with a median of ~3 ha. Though we recognize a limitation of the small sample size, these values fall within the reported home range sizes for both species (see Introduction). However, research in the same or nearby geographical locations reported substantially larger maximum home range sizes than reported here. In New Mexico, Weaver et al. (2025) tracked 8 *T. ornata* 3–7 times per week between 2020 and 2022 and reported annual 100% MCP from 0.3 ha (female) to 30.9 ha (female). The data from Weaver et al. (2025) include the three turtles reported in this study; these individuals were the only ones tracked for a full active season (May–October), enabling their use in our analysis. The exceptionally large home range size of ~31 ha at this study site was in response to high‐severity wildfire that occurred in early 2022 (Weaver et al. 2025). In North Carolina, Roe et al. (2020) tracked 57 *T. c. carolina* at two sites (100 and 160 straight line km south of our study site) over a 5‐year period, with each turtle tracked once per week during their active season obtaining ~27 location points per turtle per year. The annual 100% MCP for both sexes ranged from 0.6 ha (male) to 113.1 ha (female), while the mean was 9.5 and 17.3 ha for females and 4.7 and 9.1 ha for males, depending on the location (Roe et al. 2020). If we are making inferences based on a single location point per week, the mean home range of ~2 ha (range: 0.7–3.4 ha) for *T. c. carolina* in our study is considered two to 8 times smaller than the mean for nearby populations reported by Roe et al. (2020).

Human presence can influence animal behavior. Varty et al. (2024) used GPS loggers and explicitly investigated whether handling adult female wood turtles ( *Glyptemys insculpta* ) for 10 min affected their movement. The study found that turtles moved significantly farther after handling, particularly in the first 50 h, suggesting that handling induced a flight response in turtles. In our study, we made efforts to minimize disturbance to turtles. We handled turtles only upon first capture for taking morphometric data and attaching the transmitter. We did not handle the turtles for any subsequent relocations. We maintained a distance (~5 m) from the turtles while recording the data, and the only time we were closer was to get a precise location point, which lasted ~30 s. The entire process for recording the location and additional data usually lasted less than 3 min. When visual checks were necessary (e.g., turtles were not clearly visible), we used cautious movements and minimized human noise to avoid disturbing the turtles. Despite our best efforts to minimize disturbance, we acknowledge that our presence may have influenced box turtle behavior. Future studies should explicitly test for such effects using less intrusive methods, such as GPS tags. It is also worth noting that the *T. c. carolina* site is open to the public and regularly used for youth programs that extend beyond designated trails, suggesting that turtles at this site are likely to encounter humans often.

Although our study is the first to use data simulated from VHF radio‐tracking to understand how the number of relocation points affects the estimation of spatial metrics in turtles, our results generally corroborate a small number of studies that compared data gathered with VHF radio‐telemetry versus GPS loggers. For example, Thompson (2018) tracked 48 adult Wood Turtles with radio telemetry and 8 with GPS loggers to compare the efficiency and accuracy of each method. Radio telemetry was conducted 1–2 times per week, while GPS loggers provided a satellite fix every 4 h. The results showed that the mean annual distance moved from the radio telemetry data was significantly lower compared to the estimates using GPS loggers, with GPS loggers estimating a distance more than three times greater. GPS loggers provide additional spatial and temporal data that radio tracking lacks, such as migration corridors, nesting and staging habitats, and movement timing (Christensen and Chow‐Fraser 2014; Cain and Cross 2018). While GPS loggers are becoming more advanced and affordable, one important limiting factor for box turtles is the weight of the equipment and habitat characteristics. Box turtle adults usually weigh around 300 to 600 g (Bernstein et al. 2007; Altobelli et al. 2021), which limits the battery power that can be used and consequently the longevity of GPS loggers. Additionally, GPS loggers do not work well underwater and struggle to maintain a signal when turtles are in dense vegetation, burrows, or areas with obstructions like logs and rocks (Christensen and Chow‐Fraser 2014; Cain and Cross 2018; Hulbert et al. 2024).

Our study highlights two important points when interpreting the home range of box turtles. First, home range comparisons across studies with different tracking schedules should be taken with caution. In addition, local environmental conditions can fluctuate significantly from year to year and influence annual movement behavior, which should also be taken into consideration. Second, using radio‐telemetry studies to inform conservation strategies for a specific area should treat 100% MCP estimates as at least half of the landscape utilized by box turtles. Home range and movement information for conservation‐reliant species are often essential considerations in the development of land protection policies (Fraser et al. 2018; Crane et al. 2021). As relatively slow‐moving, box turtles are particularly vulnerable to habitat destruction, which leads to disproportionately high mortality and population fragmentation (Wingfield 2005; Brown et al. 2021). Our data indicate that turtles make occasional but substantially longer movements within a single day. These outliers demonstrate that this behavior can make box turtles more vulnerable in fragmented areas, especially urban environments and populations located near heavily trafficked roads. Understanding the minimum continuous habitat needed to reduce anthropogenic mortality is crucial for informing and improving habitat protection policies. Increasing the amount of location data can provide more comprehensive information about the habitat size necessary to support the ecological needs of specific *T. ornata* and *T. c. carolina* populations.

## Author Contributions

**Kristen E. Liles:** data curation (equal), investigation (lead), methodology (equal), validation (equal), visualization (equal), writing – original draft (lead), writing – review and editing (equal). **Kenia Barajas‐Salazar:** data curation (equal), formal analysis (equal), investigation (equal), visualization (equal), writing – original draft (equal), writing – review and editing (supporting). **Ivana Mali:** conceptualization (lead), data curation (equal), formal analysis (equal), resources (lead), supervision (lead), validation (equal), visualization (equal), writing – original draft (equal), writing – review and editing (lead).

## Conflicts of Interest

The authors declare no conflicts of interest.

## Supporting information


**Data S1:** ece372137‐sup‐0001‐DataS1.zip.

## Data Availability

The raw data and R scripts used in this manuscript are provided as Supporting Information.
